# Reconstructing Neural Circuits in 3D, Nanometer by Nanometer

**DOI:** 10.1371/journal.pbio.0020388

**Published:** 2004-10-19

Understanding how the brain processes and stores information depends in large part on knowing which neurons are involved in a particular process and how they're organized into functional networks. Each of the 10 billion or so neurons in the brain has thousands of connections to other neurons, sending (via axons) or receiving (via dendrites) the signals that allow us to think. Each neuron can transmit signals to both local and distant neurons, and it is by mapping these networks that neuroscientists can discern correlations between neural connectivity and physiological responses and ultimately unveil the computational algorithms underlying brain function. Since the beginning of cellular neuroscience at the end of the 19th century, neuronal connections have been explored by tracing axons and dendrites under the light microscope. But even with the resolution of state-of-the-art light microscopy, this approach works only if a small subset of neurons is stained and thus leaves most of the network hidden.

Electron microscopy, on the other hand, can provide the spatial resolution necessary both to resolve processes in densely packed neural “wire bundles” and to identify synapses faithfully, but individual electron microscopic images are restricted to two dimensions. Transmission electron microscopy provides cross-sectional images through tissue, while scanning electron microscopy typically provides the appearance of 3D but in reality maps only the specimen surface and is thus blind to the connections within. It's possible to wrest 3D information from the transmission electron microscope by using tilt-series tomography, but sections can't be much thicker than 1 micron (a millionth of a meter). Data from thicker volumes can be obtained, but the process so far has been so painstaking and time-intensive—it involves, among other labor-intensive tasks, manually reconstructing serial sections—that few undertake it.

It should, however, be possible to get similar data with “serial block-face imaging,” which involves repeatedly cutting section after section from a plastic-embedded block of tissue and photographing what's left. Scanning electron microscopy is needed for this task, but sample preparation methods are like those used for transmission electron microscopy, albeit with a few additional steps to enhance contrast.

**Figure:**
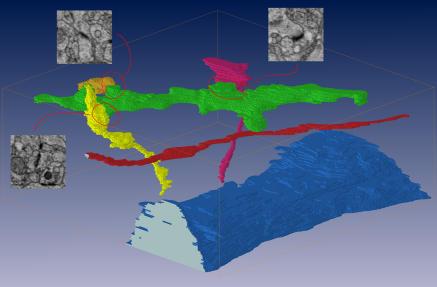
Neurite Reconstruction Manual reconstruction of selected processes in cortical tissue

This is exactly what Winfried Denk and Heinz Horstmann have done to obtain “truly 3D datasets” using a method they call “serial block-face scanning electron microscopy” (SBFSEM), for which they constructed a “microtome” that goes inside the scanning electron microscope chamber. The resolution achieved is sufficient to reveal “even the thinnest of axons” and identify synapses. The SBFSEM method can generate stacks of thousands of ultra-thin sections, 50–70 nanometers (a nanometer is a billionth of a meter) thick, generating 3D datasets to reconstruct the topology and circuitry of neurons in brain tissue.

The authors' custom-designed microtome holds the tissue block in a way that ensures image alignment and maintains focus; all the while the specimen surface is positioned close enough to the objective lens to allow high-resolution imaging.

Denk and Horstmann expect that with this method they might ultimately be able to cut sections thinner than the 50 nanometers that their current setup manages. This then would allow them to cut sections even thinner than what is routinely possible in conventional transmission electron microscopy. While the authors doubt that the lateral resolution will ever reach that of transmission electron microscopy, they also argue that such high resolution may not actually be needed to trace neuronal connectivity. On the other hand, the method accelerates 3D electron microscopic data collection “by several orders of magnitude” by obviating the need for the labor-intensive adjustments to correct alignment and distortion required by other methods, an advance that is crucial for large-volume neuroanatomy and might, in addition, open up many hitherto inaccessible problems to ultra-structural investigations.

